# In vitro screening of nutrients regulating sheep intramuscular angiogenesis, adipogenesis, and lipid deposition using an organoid model

**DOI:** 10.1186/s40104-025-01276-9

**Published:** 2025-10-27

**Authors:** Yating Li, Xiaoying Sun, Yue Lv, Jiaxin Liu, Xinyi Mao, Jinyan Yu, Yanrong Feng, Long Cheng, Yifan Xiang, Yu Xin, Zhongzuo Huang, Yichen Luo, Yan Zhang, Junxing Zhao, Bo Wang

**Affiliations:** 1 State Key Laboratory of Animal Nutrition and Feeding, College of Animal Science and Technology, China Agricultural University, Beijing, 100193 People’s Republic of China; 2 College of Animal Science, Shanxi Agricultural University, Taigu, Shanxi 030801 People’s Republic of China; 3 Yazhouwan National Laboratory, Sanya, Hainan 572024 People’s Republic of China

**Keywords:** Angiogenesis, Intramuscular fat, Meat quality, Nutrient, Sheep

## Abstract

**Background:**

The deposition of intramuscular fat (IMF) in livestock can enhance the flavor and tenderness of meat products, significantly increasing consumer satisfaction. To achieve this industrial trait, this study investigated the regulatory effects of 20 dietary nutrients on sheep IMF deposition using a 3D organoid culture model.

**Results:**

Key nutrients enhancing angiogenesis, adipocyte differentiation, and lipid accumulation were identified through assessments of capillary sprouts development, mRNA expression, and Oil Red O staining. Vitamins C (VC), E (VE), and K_1_ (VK1), guanidinoacetic acid (GAA), leucine (Leu), lysine (Lys), methionine (Met), N-carbamylglutamate (NCG), tryptophan (Trp), α-linolenic acid (ALA), linoleic acid (LA), *cis*-9, *trans*-11 conjugated linoleic acid (*c*9, *t*11-CLA), acetic acid (HAc), and sodium acetate (NaAc) stimulated while vitamins B_9_ (VB9), D (VD), K_2_ (VK2), taurine (Tau), and sodium butyrate (NaBu) inhibited angiogenesis (*P* < 0.05). Furthermore, VC, VE, VK1, VK2, GAA, Leu, NCG, Trp, ALA, LA, and HAc enhanced adipocyte differentiation, with VE, VK1, GAA, Leu, LA, and HAc additionally elevating lipid accumulation (*P* < 0.05).

**Conclusions:**

Various nutrients play distinct regulatory roles in angiogenesis, adipocyte differentiation, and lipid accumulation. These findings provide a roadmap for further optimizing the production of marbled meat through nutritional intervention in actual livestock breeding production.

**Graphical Abstract:**

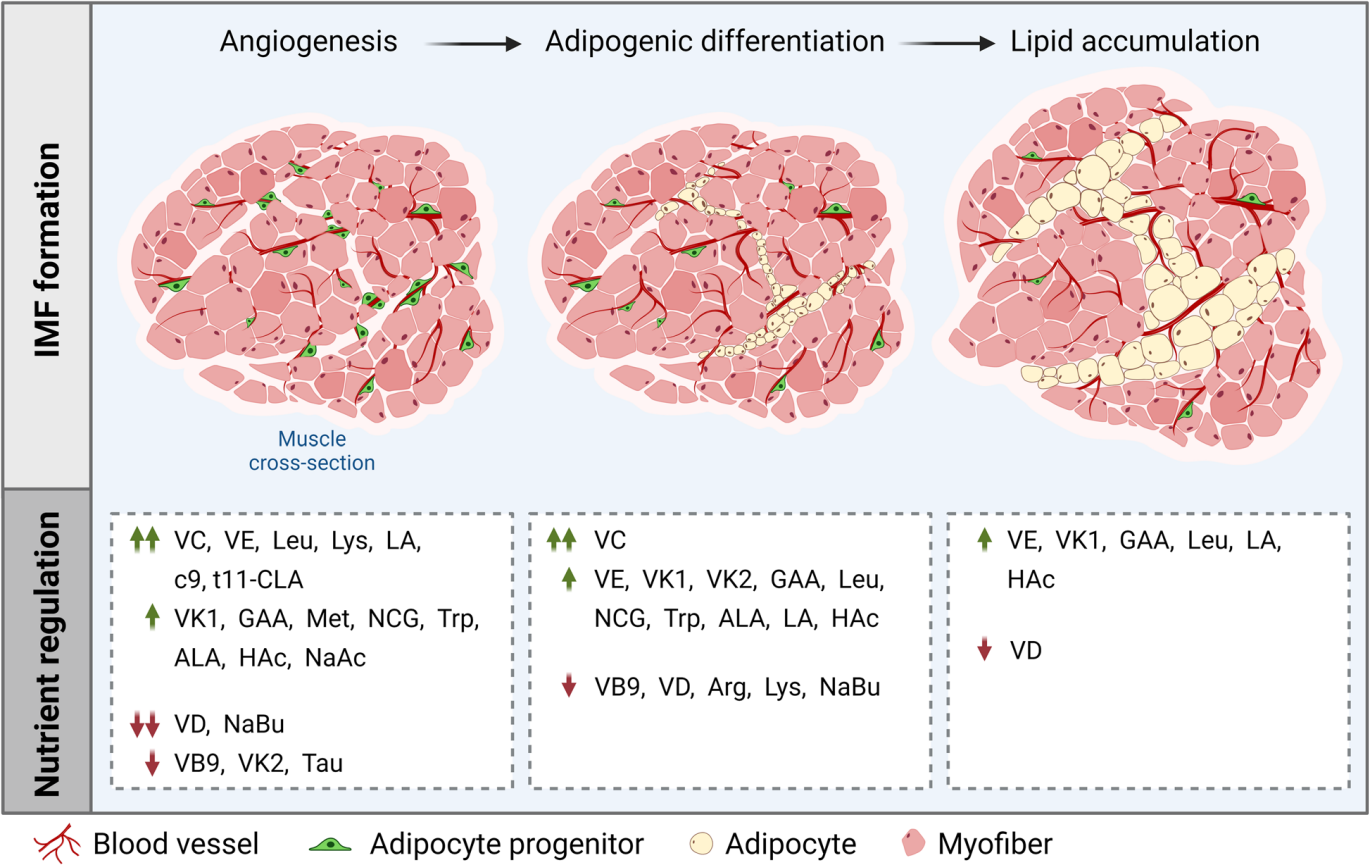

## Introduction

Intramuscular fat (IMF), also known as marbled fat, refers to the visible spots or stripes of white adipose tissue within skeletal muscles, specifically between muscle fibers. Consumer preference for marbled meat has increased due to its enhanced flavor, juiciness, and tenderness [1]. Compared to subcutaneous and visceral fat, IMF contains higher proportions of monounsaturated and polyunsaturated fatty acids [2], making it a healthier meat product. Consequently, IMF content has become an important determinant of meat quality and market value. 

In livestock production, high-energy diet feeding during the fattening stage effectively promotes IMF accumulation but often leads to excessive accumulation of undesired fat, such as visceral and subcutaneous fat. Fat tissues grow through adipocyte hyperplasia (increase in cell number) and hypertrophy (increase in cell size) [3]. Unlike subcutaneous and visceral adipocytes, which are primarily formed prenatally [4, 5] and remain constant in adipocyte number postnatally [6], intramuscular preadipocytes start to proliferate and differentiate into adipocytes around birth until late life [4, 5, 7]. Leveraging this developmental timing difference offers a promising strategy to selectively enhance IMF deposition by increasing intramuscular adipocyte number [8]. For instance, we previously demonstrated that neonatal vitamin A administration between birth and one month of age increased intramuscular adipocytes and enhanced intramuscular fat accumulation by 45% and 82% in cattle [9, 10] and sheep [11], respectively, without affecting subcutaneous and visceral fat accumulation.

Adipose tissue consists of multiple types of cells, and their interactions critically regulate adipocyte development, remodeling, migration, and death [12–15]. As a highly vascularized organ, adipose tissue depends on adequate blood supply for nutrient delivery and endothelial-derived signaling molecules that modulate adipogenesis and tissue expansion [16]. Pericytes within the perivascular matrix express the adipocyte progenitor marker and can differentiate into mature adipocytes upon PPARγ activation [17]. Increasing vascular supply and the pool of adipocyte progenitors through angiogenesis is therefore considered the rate-limiting step in adipocyte formation [18]. This is particularly relevant for IMF, where adipocytes are histologically distributed around capillaries [19], and capillary density positively correlates with marbling level in meat [20]. Stimulating angiogenesis in skeletal muscles early in life may enhance the potential for intramuscular adipocyte formation.

Most in vitro studies on adipogenesis rely on 2D cell cultures, which lack the multicellular interactions present in vivo. To address this limitation, we developed a vascularized adipose organoid model using sheep muscle-derived stromal vascular fraction (SVF) cells to simulate IMF development in vivo [21]. In this model, cell spheroids are seeded in Matrigel and undergo angiogenesis and adipogenesis. It can be used to study the interactions between vascular and adipocytes, serving as a precise tool to investigate the processes of preadipocyte commitment and terminal adipogenic differentiation.

Using this model, our next objective is to identify bioactive nutrients that regulate intramuscular adipocyte formation (hyperplasia) and lipid accumulation (hypertrophy). This aims to develop a comprehensive nutritional strategy to promote intramuscular adipocyte hyperplasia in neonates and hypertrophy in finishing-stage animals. Various nutrients that are frequently used in livestock production influence adipogenesis. In vitro studies reported that vitamin C (VC) [22] and vitamin E (VE) [23] promote, while vitamin D (VD) [24], vitamin K_2_ (VK2) [25], arginine (Arg) [26], N-carbamylglutamate (NCG) [27], α-linolenic acid (ALA) [28], and conjugated linoleic acid (CLA) [28] inhibit adipocyte differentiation. Regarding lipid accumulation, linoleic acid (LA) promotes [29], whereas VC [30], VD [24], and VE [31] inhibit it. Lysine (Lys) [32] and methionine (Met) [33] deficiency also inhibit both adipocyte differentiation and lipid accumulation. In vivo experiments found that fat deposition of livestock was reduced by supplementing with Arg [34], guanidinoacetic acid (GAA) [35, 36], and acetic acid (HAc) [37–39]. However, these findings primarily derive from 2D cultures of 3T3-L1 cells or primary adipocytes, often from non-intramuscular depots. Furthermore, livestock nutrition studies have largely focused on nutrient effects during fattening, neglecting early developmental stages. Therefore, we selected 20 relevant nutrients potentially affecting IMF deposition and studied their roles in intramuscular angiogenesis, adipogenesis, and lipid accumulation using our 3D organoid model. Key pro-adipogenic nutrients identified through this approach, potentially after encapsulation for targeted delivery, could be supplemented to enhance IMF deposition in livestock.

## Materials and methods

### Cell isolation from tissue

Healthy 1-day-old newborn Hu sheep were transported to the laboratory, then euthanized through anesthesia and exsanguination. This procedure followed the Guidelines for the Care and Use of Laboratory Animals in China and was approved by the Ethics Committee of China Agricultural University (Approval No. AW60604202-1-1). A piece of the longissimus dorsi muscle was isolated and washed twice in PBS buffer. A 1-cm^3^ muscle block was quickly cut into small pieces with scissors in 2 mL Dulbecco's Modified Eagle Medium (DMEM) containing 100 U/mL collagenase Ι (Gibco, NY, USA), 0.1 mg/mL DNase Ι (Aladdin, Shanghai, China), 2 mg/mL dispase Ⅱ (Beyotime, Shanghai, China), and 3 μmol/L CaCl_2_. The tissue suspension was incubated in a 37 °C water bath and was blown with a pipette every 5 min to fully digest the tissue. Individual muscle-derived stromal vascular fraction (SVF) cells were obtained after incubating for 30 min. Digestion was terminated by adding 2 mL DMEM containing 10% FBS (Beyotime, Shanghai, China). The suspension was filtered through a 100-μm filter to remove tissue debris and then centrifuged at 800 × *g* for 5 min to obtain cell precipitation. The cells were resuspended in DMEM containing 10% FBS, 100 IU/mL penicillin, and 100 mg/mL streptomycin (Beyotime, Shanghai, China) and then transferred to a 10-cm cell culture dish in a 37 °C, 5% CO_2_ humidified incubator.

### Cell culture

Adipogenic differentiation of muscle-derived SVF cells was induced using our previously developed 3D cell culture model [21], simulating the process of IMF formation in sheep. Briefly, the isolated SVF cells were resuspended in endothelial basal medium (EBM) containing 10% FBS, 100 IU/mL penicillin, and 100 mg/mL streptomycin after 48 h of proliferation. The cells were counted using a hemocytometer and diluted appropriately. A volume of 25 μL of cell suspension was dropped onto the inner side of the lid of a 10-cm cell culture dish, making the droplets hang on the lid, with 2× 10^5^ cells in each droplet. Meanwhile, 3 mL PBS was added to the bottom of the dish to enhance the humidity. The cell culture dish was placed in a humidified incubator for 3 d. The cells in the droplet would settle to the bottom of the droplet due to gravity and gradually form a 3D cell spheroid. A pre-cooled 96-well cell culture plate was coated with 50 μL 5 mg/mL Matrigel basement membrane matrix (Corning, NY, USA) into each well and then placed at 37 °C for 10 min to solidify the Matrigel matrix. Three 3D cell spheroids were transferred to each well of the coated 96-well cell culture plate and cultured in EBM containing 10% FBS, 100 IU/mL penicillin, and 100 mg/mL streptomycin for 6 d. The cell spheroids grew dense vascular endothelial cells and radially extended into the Matrigel matrix. Then, the cells differentiated into adipocytes in DMEM supplemented with 0.5 mmol/L IBMX (Beyotime, Shanghai, China), 1 µmol/L DEX (Beyotime, Shanghai, China), 10 µg/mL insulin (Beyotime, Shanghai, China), and 125 µmol/L indomethacin (Aladdin, Shanghai, China) for 6 d and accumulated lipid droplets in DMEM supplemented with 5 µg/mL insulin (Beyotime, Shanghai, China) for 12 d.

**Fig. 1:**
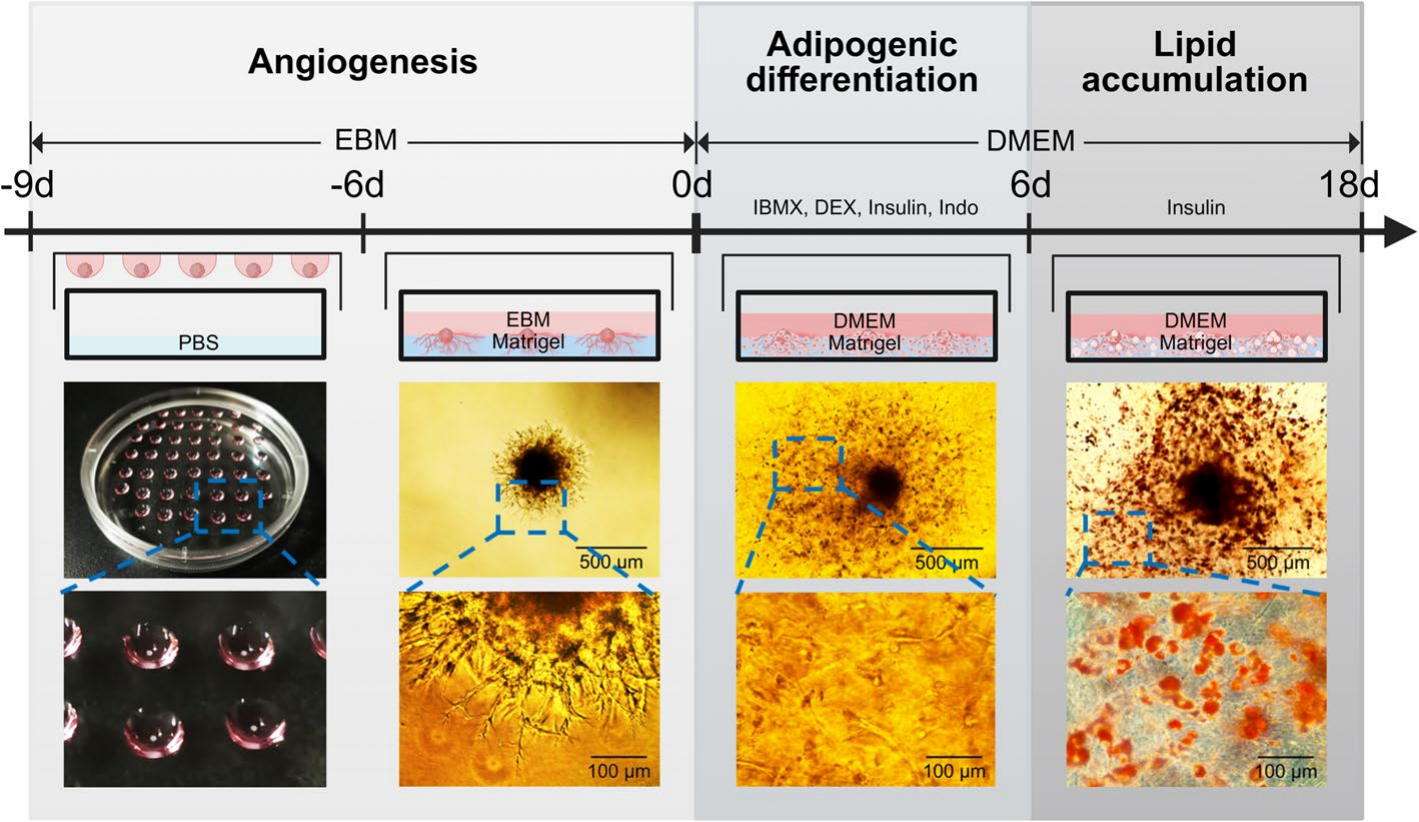
3D cell culture process of IMF organoid. Cell spheres are formed in hanging drops (−9 to −6 d). Cell spheres are transferred to Matrigel coated wells and develop vascular sprouts in EBM (−6 to 0 d). Adipogenic differentiation of cells is induced by 0.5 mmol/L IBMX, 1 μmol/L DEX, 10 μg/mL insulin, and 125 μmol/L indomethacin for 6 days (0 to 6 d) and maintained in DMEM supplemented with 5 μg/mL insulin for lipid droplet formation. DMEM: Dulbecco's Modified Eagle Medium; EBM: Endothelial Basal Medium

As illustrated in Fig. 1, the 20 nutrients tested in this study were added at three different stages of the cell culture process: angiogenesis (−9 to 0 d), adipogenic differentiation (0 to 6 d), and lipid accumulation (6 to 18 d). Each nutrient was added to the cell culture medium at low and high doses. The dosages were based on numerous cell experiment literature. Meanwhile, to facilitate the assessment of the applicability of these concentrations in vivo, Table 1 summarizes the actual detected concentrations of the 20 nutrients in animal blood. The VC, leucine (Leu), and tryptophan (Trp) used in this study were purchased from Beyotime Biotechnology (Shanghai, China), and the remaining 17 nutrients were all purchased from Aladdin Scientific Corp. (Shanghai, China). Considering that the cells may be affected by the culture conditions and operation variations of investigators, cell culture, nutrient treatment, and Oil Red O staining of lipid droplets were repeated 3 times on different days using cells from different animals.

**Table 1:** Comparison of nutrient concentrations in this cell experiment and animal blood

Nutrients		Concentration in this in vitro research	Concentration ranges in animal blood	Species	Reference
Vitamins	VB9	10, 100 μmol/L	1.59–51.13 nmol/L	Sheep	[40–43]
	VC	50, 200 μmol/L	69.30–78.90 μmol/L	Sheep	[44]
	VD (1,25-VD)	1, 10 nmol/L	43.40–143.00 pmol/L	Sheep	[45, 46]
	VE (α-tocopherol)	5, 50 μmol/L	0.23–8.13 μmol/L	Sheep	[47, 48]
	VK1	10, 100 μmol/L	0–1.00 μmol/L	Human	[49]
	VK2	10, 100 μmol/L	0.10–0.50 nmol/L	Human	[50]
Amino acids (derivatives)	Arg	0.1, 1 mmol/L	137.00–353.00 μmol/L	Sheep	[51–53]
	GAA	0.2, 2 mmol/L	1.00–250.56 μmol/L	Sheep	[54, 55]
	Leu	0.5, 2 mmol/L	52.50–381.18 μmol/L	Sheep	[56, 57]
	Lys	0.1, 1 mmol/L	147.00–410.42 μmol/L	Sheep	[53, 56]
	Met	0.1, 1 mg/mL	2.19–22.98 μg/mL	Sheep	[51, 53]
	NCG	0.1, 1 mmol/L	0–3.22 μmol/L	Pig	[58]
	Tau	0.5, 5 mmol/L	239.71–399.52 μmol/L	Sheep	[56]
	Trp	0.1, 1 mmol/L	19.00–56.90 μmol/L	Sheep	[52]
Fatty acids (salts)	ALA	10, 100 μmol/L	56.12–144.32 μmol/L	Sheep	[59]
	LA	5, 50 μmol/L	464.00–987.43 μmol/L	Sheep	[59]
	*c*9, *t*11-CLA	100, 200 μmol/L	4.64–73.61 μmol/L	Sheep	[59]
	HAc	0.2, 2 mmol/L	0.19–0.89 mmol/L	Sheep	[60]
	NaAc	0.5, 5 mmol/L	0.41–2.80 mmol/L (acetate)	Sheep	[61–63]
	NaBu	50, 500 μmol/L	11.00–19.00 μmol/L (butyrate)	Sheep	[61]

### Length measurement of vascular sprouts

After different nutrients were added to the angiogenesis stage, cells were photographed under a microscope (Invitrogen, Carlsbad, USA) at 0 d in Fig. 1. The longest diameter passing through the center of the cell sphere was measured using Image-Pro Plus 6.0 (Media Cybernetics, MD, USA) to evaluate the length of the vascular sprouts.

### qRT-PCR

In the 96-well cell culture plate, add 100 μL of TRIzol into each well to dissolve the Matrigel matrix and extract total RNA from the cells. Total RNA was isolated by chloroform and isopropanol sequentially. The concentration and 260/280 ratio of total RNA were measured using a NanoDrop-1000 Spectrophotometer (Nanodrop, Wilmington, USA) to ensure that the total RNA was pure and undegraded. Then, the total RNA was reverse transcribed into cDNA using the *Evo M-MLV* RT Mix Kit (Accurate, Hunan, China). A 10 μL reaction system, including 5 μL mix, 2 μL primer, 2 μL cDNA, and 1 μL H_2_O, underwent 40 thermal cycles at 95 °C for 15 s, 60 °C for 30 s, and 72 °C for 30 s. Eventually, the melting peak of the genes was observed, and the relative mRNA expression was calculated by 2^−△△Ct^ [64]. Table 2 displays the primer sequences of the gene.

**Table 2:** Primer sequences for qRT-PCR

Genes	Sequence (5'→3')	Length, bp
*FGF2*	F: TCAAGCAGAAGAGAGAGGGGTT	202
	R: CCCAGTTCGTTTCAGTGCC	
*VEGFA*	F: TCACCAAAGCCAGCACATAGGA	107
	R: GCCTCGGCTTGTCACATTTTTC	
*VEGFR1*	F: AAGGAGATACGCGACTTCCTCTG	194
	R: CCGCATCCTCATCCCATCCTT	
*VEGFR2*	F: TCTGCCTACCTCACCTGTTTCC	278
	R: TCAGTCCACTAAAAGATGGGGC	
*PPARγ*	F: GCCGGGTCTGTGGGGATAAA	220
	R: CGCCCAAACCTGATGGCATT	
*ZFP423*	F: TCGTCGGATGTGGAGGTGTCTTC	111
	R: GTTGGAGAACTTGAGGTCGCACTG	
*CEBPA*	F: ATCAGCGCCTACATCGACCC	135
	R: GCCCGGGTAGTCAAAGTCGT	
*CEBPB*	F: AGCAAGGCCAAGAAGACGGT	213
	R: GAACAAGTTCCGCAGGGTGC	
*FABP4*	F: TGATTACATGAAAGAAGTGGGTGTGG	115
	R: GTGCTTTCTGATTATGTTGACCACAT	
*18S*	F: CCTGCGGCTTAATTTGACTC	118
	R: AACTAAGAACGGCCATGCAC	

### Oil Red O staining

After 12 d of adipogenic maintenance, a large number of lipid droplets were deposited in the cells under the microscope. Added 200 μL of 4% paraformaldehyde to each well of the 96-well cell culture plate to dissolve the Matrigel matrix and fix the cell morphology. The cells were stained with 60% Oil Red O solution for 30 min, washed with 50% isopropyl alcohol, and then soaked in PBS for observation and photography under a microscope. Finally, the Oil Red O staining solution in the cells was extracted with pure isopropyl alcohol, and the absorbance of the staining solution was detected at the 510 nm wavelength using a microplate reader (BioTek, VT, USA).

### Statistical analysis

Data was analyzed using one-way analysis of variance and Duncan multiple comparison in SPSS 27.0 (IBM, NY, USA). A *P* < 0.05 indicates a statistical difference.

## Results

### Effects of different nutrients on angiogenesis of sheep muscle-derived SVF cells

By adding nutrients during the angiogenesis stage (−9 to 0 d, Fig. 1), the growth of cell spheroid vascular sprouts was promoted by eleven nutrients, including VC, VE, vitamin K_1_ (VK1), GAA, Leu, Lys, Met, Trp, ALA, LA, and *cis*-9, *trans*-11 conjugated linoleic acid (*c*9, *t*11-CLA), while inhibited by VD, VK2, and sodium butyrate (NaBu) (Table 3). Meanwhile, supplementation of VE, Leu, NCG, *c*9, *t*11-CLA, and HAc during the angiogenesis stage increased lipid deposition in mature adipocytes, while taurine (Tau) and NaBu decreased the amount of lipid droplet (*P* < 0.05) (Table 4). In addition, supplementation of VC, VE, Leu, Lys, LA, *c*9, *t*11-CLA, and sodium acetate (NaAc) at the angiogenic stage upregulated the mRNA expression of angiogenesis-related genes, including *FGF2*, *VEGFA*, and *VEGFR2,* to promote angiogenesis. Vitamin B_9_ (VB9) upregulated *VEGFR1* (negative regulation gene), while VD and NaBu downregulated *VEGFR2* to inhibit angiogenesis (*P* < 0.05) (Table 5). Based on these results, VC, VE, Leu, Lys, LA, and *c*9, *t*11-CLA strongly promote the angiogenesis of SVF cells.

**Table 3:** Length of vascular sprouts after nutrient treatment during the angiogenesis stage

Nutrients		Control	Low dose	High dose	SEM	*P*-value
Vitamins	VB9	1.000	1.002	1.046	0.0123	0.238
	VC	1.000^b^	1.253^a^	1.332^a^	0.0308	< 0.001
	VD (1,25-VD)	1.000^a^	0.938^b^	0.937^b^	0.0819	< 0.001
	VE (α-tocopherol)	1.000^b^	1.058^ab^	1.120^a^	0.0174	0.007
	VK1	1.000^b^	1.038^ab^	1.071^a^	0.0629	0.043
	VK2	1.000^a^	0.970^a^	0.751^b^	0.0239	< 0.001
Amino acids (derivatives)	Arg	1.000	0.982	0.978	0.0169	0.868
	GAA	1.000^c^	1.092^b^	1.201^a^	0.0182	< 0.001
	Leu	1.000^c^	1.066^b^	1.171^a^	0.0161	< 0.001
	Lys	1.000^b^	1.249^a^	1.302^a^	0.0525	0.034
	Met	1.000^b^	1.068^ab^	1.105^a^	0.0154	0.010
	NCG	1.000	1.075	1.080	0.0167	0.079
	Tau	1.000	0.977	0.963	0.0161	0.617
	Trp	1.000^b^	1.027^b^	1.125^a^	0.0161	0.002
Fatty acids (salts)	ALA	1.000^b^	0.987^b^	1.162^a^	0.0274	0.023
	LA	1.000^b^	1.127^a^	1.230^a^	0.0274	< 0.001
	*c*9, *t*11-CLA	1.000^b^	0.959^b^	1.098^a^	0.0211	0.013
	HAc	1.000	1.110	1.015	0.0215	0.102
	NaAc	1.000	1.021	0.954	0.0166	0.241
	NaBu	1.000^a^	1.037^a^	0.753^b^	0.0243	< 0.001

^a,b^ Means with different letters are significantly different from each other (*P* < 0.05)

**Table 4:** The OD values of Oil Red O staining in mature cells treated with nutrients during the angiogenesis stage

Nutrients		Control	Low dose	High dose	SEM	*P*-value
Vitamins	VB9	1.000	1.253	1.003	0.0027	0.608
	VC	1.000	1.629	1.30	0.0035	0.183
	VD (1,25-VD)	1.000	0.920	1.033	0.0145	0.714
	VE (α-tocopherol)	1.000^b^	0.965^b^	1.122^a^	0.0044	0.045
	VK1	1.000	0.887	0.865	0.0075	0.460
	VK2	1.000	1.088	1.233	0.0020	0.149
Amino acids (derivatives)	Arg	1.000	1.113	1.224	0.0025	0.523
	GAA	1.000	0.984	0.929	0.0022	0.235
	Leu	1.000^b^	1.202^ab^	1.546^a^	0.6579	0.042
	Lys	1.000	1.062	1.226	0.0085	0.255
	Met	1.000	1.137	1.217	0.0019	0.157
	NCG	1.000^b^	1.536^a^	1.100^ab^	0.0033	0.048
	Tau	1.000^a^	0.830^b^	0.873^b^	0.0032	0.045
	Trp	1.000	0.704	0.591	0.0132	0.529
Fatty acids (salts)	ALA	1.000	1.134	1.172	0.0064	0.112
	LA	1.000	1.125	1.099	0.0072	0.103
	*c*9, *t*11-CLA	1.000^b^	1.063^b^	1.278^a^	0.0152	0.014
	HAc	1.000^b^	1.071^b^	1.387^a^	0.0044	0.031
	NaAc	1.000	1.010	0.938	0.0018	0.732
	NaBu	1.000^a^	0.663^b^	0.618^b^	0.0106	0.009

^a,b^ Means with different letters are significantly different from each other (*P* < 0.05)

**Table 5:** Expression of angiogenesis-related genes in cells treated with nutrients

Nutrients		Genes	Control	Low dose	High dose	SEM	*P*-value
Vitamins	VB9	*FGF2*	1.000	0.431	0.527	0.1332	0.140
		*VEGFA*	1.000	1.646	1.512	0.1540	0.197
		*VEGFR1*	1.000^b^	6.466^a^	5.071^a^	1.2163	< 0.001
		*VEGFR2*	1.000	0.756	0.610	0.1837	0.631
	VC	*FGF2*	1.000	1.210	1.494	0.2165	0.668
		*VEGFA*	1.000	0.576	0.556	0.1017	0.077
		*VEGFR1*	1.000	1.484	1.768	0.2184	0.384
		*VEGFR2*	1.000^b^	1.377^a^	1.372^a^	0.0863	0.030
	VD (1,25-VD)	*FGF2*	1.000	1.355	1.576	0.1503	0.332
		*VEGFA*	1.000	1.478	2.553	0.3610	0.055
		*VEGFR1*	1.000	0.774	1.183	0.1797	0.698
		*VEGFR2*	1.000^a^	0.351^b^	0.094^c^	0.1218	< 0.001
	VE (α-tocopherol)	*FGF2*	1.000	1.370	1.471	0.1069	0.151
		*VEGFA*	1.000^b^	1.417^ab^	1.796^a^	0.1319	0.018
		*VEGFR1*	1.000^b^	3.248^a^	2.442^ab^	0.3170	0.025
		*VEGFR2*	1.000	1.268	0.929	0.1149	0.696
	VK1	*FGF2*	1.000	1.265	1.066	0.1178	0.664
		*VEGFA*	1.000	1.391	2.187	0.2461	0.109
		*VEGFR1*	1.000	0.692	0.451	0.1467	0.197
		*VEGFR2*	1.000	1.178	0.998	0.0691	0.500
	VK2	*FGF2*	1.000	0.729	0.830	0.0682	0.268
		*VEGFA*	1.000	1.461	1.508	0.1237	0.167
		*VEGFR1*	1.000	0.773	1.271	0.1165	0.164
		*VEGFR2*	1.000	0.774	0.731	0.0582	0.112
Amino acids (derivatives)	Arg	*FGF2*	1.000	0.839	1.203	0.1268	0.544
		*VEGFA*	1.000	0.957	1.296	0.1183	0.482
		*VEGFR1*	1.000	0.911	1.259	0.2168	0.842
		*VEGFR2*	1.000	0.878	0.964	0.0835	0.855
	GAA	*FGF2*	1.000	1.012	0.823	0.0619	0.411
		*VEGFA*	1.000	0.827	1.104	0.0428	0.123
		*VEGFR1*	1.000	0.784	0.951	0.1269	0.801
		*VEGFR2*	1.000	0.870	0.565	0.0570	0.200
	Leu	*FGF2*	1.000	1.143	1.343	0.0817	0.215
		*VEGFA*	1.000^b^	1.585^a^	1.595^a^	0.1242	0.036
		*VEGFR1*	1.000^b^	0.981^b^	2.078^a^	0.1685	0.013
		*VEGFR2*	1.000	0.827	0.773	0.0827	0.492
	Lys	*FGF2*	1.000^b^	1.349^b^	2.590^a^	0.3137	0.039
		*VEGFA*	1.000	1.542	1.764	0.1569	0.096
		*VEGFR1*	1.000	1.524	1.551	0.2782	0.667
		*VEGFR2*	1.000	2.213	3.357	0.2526	0.090
	Met	*FGF2*	1.000	0.967	1.313	0.0800	0.144
		*VEGFA*	1.000	1.326	1.433	0.1153	0.298
		*VEGFR1*	1.000	0.642	0.438	0.1330	0.364
		*VEGFR2*	1.000	1.118	1.102	0.0811	0.817
	NCG	*FGF2*	1.000	0.629	1.021	0.1366	0.439
		*VEGFA*	1.000	0.708	0.926	0.0740	0.250
		*VEGFR1*	1.000	0.860	0.970	0.1568	0.953
		*VEGFR2*	1.000	1.125	0.718	0.0955	0.235
	Tau	*FGF2*	1.000	0.822	0.841	0.0864	0.661
		*VEGFA*	1.000	0.942	1.073	0.0686	0.764
		*VEGFR1*	1.000	0.782	0.784	0.1889	0.851
		*VEGFR2*	1.000	1.262	0.930	0.2688	0.849
	Trp	*FGF2*	1.000	0.437	0.933	0.1325	0.167
		*VEGFA*	1.000	0.699	1.089	0.1290	0.455
		*VEGFR1*	1.000	3.313	1.339	0.6444	0.150
		*VEGFR2*	1.000	0.926	1.347	0.1043	0.471
Fatty acids (salts)	ALA	*FGF2*	1.000	0.867	1.003	0.1155	0.876
		*VEGFA*	1.000	1.033	1.150	0.0293	0.074
		*VEGFR1*	1.000	1.517	1.636	0.2618	0.517
		*VEGFR2*	1.000	1.295	1.462	0.0496	0.117
	LA	*FGF2*	1.000	1.058	0.615	0.1001	0.094
		*VEGFA*	1.000^b^	1.481^b^	3.098^a^	0.2613	0.028
		*VEGFR1*	1.000^b^	5.056^a^	3.448^a^	0.6957	0.010
		*VEGFR2*	1.000	1.018	1.304	0.0685	0.264
	*c*9, *t*11-CLA	*FGF2*	1.000	1.317	1.332	0.1772	0.657
		*VEGFA*	1.000^b^	1.736^a^	1.566^ab^	0.1387	0.034
		*VEGFR1*	1.000	1.057	0.635	0.1270	0.299
		*VEGFR2*	1.000	0.950	1.079	0.0964	0.884
	HAc	*FGF2*	1.000	0.800	0.962	0.1361	0.818
		*VEGFA*	1.000	0.964	2.036	0.2888	0.202
		*VEGFR1*	1.000	0.801	0.790	0.1545	0.820
		*VEGFR2*	1.000	0.982	0.859	0.0689	0.694
	NaAc	*FGF2*	1.000^b^	1.624^a^	1.401^a^	0.0975	0.010
		*VEGFA*	1.000^b^	2.790^a^	1.561^b^	0.2664	0.003
		*VEGFR1*	1.000	1.058	0.757	0.2745	0.857
		*VEGFR2*	1.000	1.152	1.340	0.0630	0.066
	NaBu	*FGF2*	1.000^b^	0.724^b^	2.136^a^	0.2257	0.002
		*VEGFA*	1.000^b^	0.797^b^	1.426^a^	0.1010	0.012
		*VEGFR1*	1.000^b^	1.616^ab^	2.581^a^	0.2657	0.112
		*VEGFR2*	1.000^a^	0.461^b^	0.607^ab^	0.1407	0.046

^a,b^ Means with different letters are significantly different from each other (*P* < 0.05)

### Effects of different nutrients on adipogenic differentiation of sheep muscle-derived SVF cells

The VC supplementation at the adipogenic differentiation stage (0 to 6 d, Fig. 1) significantly increased lipid deposition in intramuscular adipocytes (*P* < 0.05, Table 6). In addition, supplementation of VC, VE, VK1, VK2, GAA, Leu, NCG, Trp, ALA, LA, and HAc at this stage upregulated the mRNA expression of adipocyte differentiation-related genes, including *PPARγ*, *ZFP423*, *CEBPA*, and *CEBPB*, while supplementation of VB9, VD, Arg, Lys, and NaBu inhibited it (*P* < 0.05, Table 7). According to these results, intramuscular adipocyte differentiation is strongly promoted by VC.

**Table 6:** The OD values of Oil Red O staining in mature cells treated with nutrients during the adipogenic differentiation stage

Nutrients		Control	Low dose	High dose	SEM	*P*-value
Vitamins	VB9	1.000	0.895	0.824	0.0053	0.186
	VC	1.000^b^	1.148^a^	1.250^a^	0.0046	0.037
	VD (1,25-VD)	1.000	0.835	1.032	0.0104	0.360
	VE (α-tocopherol)	1.000	1.243	1.119	0.0083	0.287
	VK1	1.000	0.895	0.848	0.0114	0.717
	VK2	1.000	0.915	1.015	0.0056	0.559
Amino acids (derivatives)	Arg	1.000	1.050	1.056	0.0069	0.813
	GAA	1.000	1.105	1.109	0.0021	0.324
	Leu	1.000	0.863	0.963	0.0065	0.706
	Lys	1.000	0.938	0.898	0.0054	0.478
	Met	1.000	0.973	1.171	0.0071	0.245
	NCG	1.000	0.894	0.861	0.0076	0.687
	Tau	1.000	0.898	0.938	0.0064	0.349
	Trp	1.000	0.939	0.896	0.0047	0.635
Fatty acids (salts)	ALA	1.000	1.102	1.132	0.0077	0.657
	LA	1.000	0.981	1.157	0.0095	0.318
	*c*9, *t*11-CLA	1.000	0.967	1.014	0.0067	0.876
	HAc	1.000	0.980	0.939	0.0039	0.744
	NaAc	1.000	0.804	0.979	0.0087	0.448
	NaBu	1.000	0.974	0.980	0.0116	0.913

^a,b^ Means with different letters are significantly different from each other (*P* < 0.05)

**Table 7:** Expression of adipogenic differentiation-related genes in cells treated with nutrients

Nutrients		Genes	Control	Low dose	High dose	SEM	*P*-value
Vitamins	VB9	*PPARγ*	1.000^a^	0.517^b^	0.860^ab^	0.0874	0.048
		*ZFP423*	1.000	1.219	0.815	0.0862	0.161
		*CEBPA*	1.000^a^	0.569^b^	0.842^ab^	0.0733	0.032
		*CEBPB*	1.000	0.787	0.924	0.0609	0.387
	VC	*PPARγ*	1.000	1.403	1.269	0.1476	0.507
		*ZFP423*	1.000^b^	1.843^a^	2.035^a^	0.1712	0.014
		*CEBPA*	1.000	1.263	1.602	0.1497	0.513
		*CEBPB*	1.000	1.054	0.936	0.0730	0.844
	VD (1,25-VD)	*PPARγ*	1.000^a^	0.569^b^	0.604^b^	0.1067	0.046
		*ZFP423*	1.000	0.941	1.147	0.0806	0.582
		*CEBPA*	1.000	0.866	0.776	0.1201	0.750
		*CEBPB*	1.000	0.905	1.078	0.0534	0.444
	VE (α-tocopherol)	*PPARγ*	1.000^b^	0.747^b^	2.180^a^	0.2199	0.003
		*ZFP423*	1.000	1.205	1.396	0.0799	0.117
		*CEBPA*	1.000^b^	0.768^b^	2.095^a^	0.2346	0.019
		*CEBPB*	1.000^b^	1.290^ab^	1.535^a^	0.0947	0.042
	VK1	*PPARγ*	1.000^b^	1.312^b^	4.202^a^	0.5550	0.016
		*ZFP423*	1.000	1.073	0.835	0.0751	0.454
		*CEBPA*	1.000^b^	1.620^ab^	2.170^a^	0.1965	0.025
		*CEBPB*	1.000	1.249	0.856	0.1020	0.302
	VK2	*PPARγ*	1.000^b^	1.588^ab^	1.929^a^	0.1651	0.048
		*ZFP423*	1.000	0.681	0.976	0.0822	0.223
		*CEBPA*	1.000	0.939	0.966	0.1008	0.975
		*CEBPB*	1.000	1.172	1.377	0.0755	0.133
Amino acids (derivatives)	Arg	*PPARγ*	1.000	0.940	0.999	0.0718	0.933
		*ZFP423*	1.000^a^	0.746^b^	0.688^b^	0.0542	0.021
		*CEBPA*	1.000	2.134	1.712	0.4312	0.440
		*CEBPB*	1.000^a^	0.902^a^	0.612^b^	0.0632	0.013
	GAA	*PPARγ*	1.000^b^	1.203^b^	3.142^a^	0.3603	0.009
		*ZFP423*	1.000	1.023	1.026	0.0672	0.988
		*CEBPA*	1.000	1.280	1.422	0.1090	0.304
		*CEBPB*	1.000	0.798	0.910	0.0649	0.489
	Leu	*PPARγ*	1.000^b^	2.359^b^	7.498^a^	1.0743	0.008
		*ZFP423*	1.000	1.312	1.411	0.1292	0.396
		*CEBPA*	1.000	0.668	1.125	0.1017	0.159
		*CEBPB*	1.000	1.140	1.624	0.1304	0.089
	Lys	*PPARγ*	1.000^a^	0.421^b^	0.439^b^	0.1224	0.036
		*ZFP423*	1.000	0.847	0.966	0.0467	0.409
		*CEBPA*	1.000	0.991	0.953	0.0389	0.892
		*CEBPB*	1.000	1.230	1.114	0.0954	0.642
	Met	*PPARγ*	1.000	0.885	0.622	0.0988	0.304
		*ZFP423*	1.000	0.843	1.139	0.0768	0.319
		*CEBPA*	1.000	1.262	0.667	0.1594	0.342
		*CEBPB*	1.000	0.782	0.764	0.0676	0.310
	NCG	*PPARγ*	1.000	1.640	2.208	0.2364	0.123
		*ZFP423*	1.000	0.774	0.793	0.0872	0.547
		*CEBPA*	1.000^b^	0.728^b^	2.075^a^	0.1765	0.006
		*CEBPB*	1.000	0.673	0.962	0.0859	0.253
	Tau	*PPARγ*	1.000	0.931	1.195	0.1456	0.755
		*ZFP423*	1.000	1.107	1.051	0.0606	0.799
		*CEBPA*	1.000	0.828	0.925	0.1661	0.928
		*CEBPB*	1.000	1.167	1.308	0.0737	0.239
	Trp	*PPARγ*	1.000	1.090	1.900	0.1769	0.080
		*ZFP423*	1.000^b^	1.117^ab^	1.584^a^	0.1074	0.044
		*CEBPA*	1.000	0.731	0.876	0.2799	0.436
		*CEBPB*	1.000	1.072	1.174	0.0644	0.588
Fatty acids (salts)	ALA	*PPARγ*	1.000^b^	0.980^b^	2.270^a^	0.2581	0.021
		*ZFP423*	1.000	0.976	1.318	0.0705	0.067
		*CEBPA*	1.000^b^	0.857^b^	1.820^a^	0.1544	0.004
		*CEBPB*	1.000	1.026	1.538	0.1116	0.060
	LA	*PPARγ*	1.000	0.851	1.047	0.1054	0.745
		*ZFP423*	1.000^b^	0.911^b^	1.343^a^	0.0703	0.010
		*CEBPA*	1.000	0.963	0.968	0.1417	0.994
		*CEBPB*	1.000	0.995	1.136	0.0548	0.536
	*c*9, *t*11-CLA	*PPARγ*	1.000	0.605	0.665	0.1208	0.336
		*ZFP423*	1.000	0.716	0.942	0.0789	0.315
		*CEBPA*	1.000	0.726	0.984	0.1452	0.597
		*CEBPB*	1.000	1.150	0.899	0.0733	0.397
	HAc	*PPARγ*	1.000^b^	0.679^b^	2.174^a^	0.3505	0.031
		*ZFP423*	1.000	0.982	0.918	0.0855	0.933
		*CEBPA*	1.000	1.112	0.869	0.1181	0.743
		*CEBPB*	1.000	0.920	1.211	0.0826	0.372
	NaAc	*PPARγ*	1.000	1.195	1.138	0.0652	0.472
		*ZFP423*	1.000	1.296	1.310	0.0739	0.138
		*CEBPA*	1.000	1.546	1.778	0.1470	0.070
		*CEBPB*	1.000	0.984	1.353	0.1374	0.508
	NaBu	*PPARγ*	1.000^a^	0.347^b^	0.254^b^	0.1157	0.001
		*ZFP423*	1.000^a^	0.620^ab^	0.284^b^	0.1132	0.002
		*CEBPA*	1.000	0.869	1.495	0.2105	0.415
		*CEBPB*	1.000	1.352	1.705	0.1141	0.074

^a,b^ Means with different letters are significantly different from each other (*P* < 0.05)

### Effects of different nutrients on lipid accumulation of sheep muscle-derived SVF cells

During the lipid droplet deposition stage (6 to 18 d, Fig. 1), supplementation of VE, VK1, Leu, and HAc significantly increased lipid deposition in intramuscular adipocytes (*P* < 0.05, Table 8). Moreover, GAA and LA upregulated the mRNA expression of lipid accumulation-related genes, including *PPARγ* and *FABP4*, while VD downregulated it (*P* < 0.05, Table 9). Overall, VE, VK1, GAA, Leu, LA, and HAc promote lipid accumulation in intramuscular adipocytes, whereas VD inhibits lipid accumulation in intramuscular adipocytes.

**Table 8:** The OD values of Oil Red O staining in mature cells treated with nutrients during the lipid accumulation stage

Nutrients		Control	Low dose	High dose	SEM	*P*-value
Vitamins	VB9	1.000	0.883	0.995	0.0008	0.513
	VC	1.000	1.380	1.216	0.0019	0.100
	VD (1,25-VD)	1.000	0.872	0.936	0.0088	0.071
	VE (α-tocopherol)	1.000^b^	1.216^ab^	1.470^a^	0.0087	0.025
	VK1	1.000^b^	1.174^b^	1.660^a^	0.0149	0.017
	VK2	1.000	1.052	1.050	0.0037	0.850
Amino acids (derivatives)	Arg	1.000	0.902	1.070	0.0074	0.202
	GAA	1.000	0.959	1.205	0.0016	0.294
	Leu	1.000^b^	1.514^b^	2.124^a^	0.0019	0.010
	Lys	1.000	1.050	1.055	0.0117	0.592
	Met	1.000	0.891	0.924	0.0034	0.307
	NCG	1.000	1.031	0.835	0.0051	0.123
	Tau	1.000	0.808	0.877	0.0091	0.170
	Trp	1.000	1.075	1.158	0.0037	0.272
Fatty acids (salts)	ALA	1.000	1.010	1.010	0.0109	0.989
	LA	1.000	0.988	1.022	0.0074	0.967
	*c*9, *t*11-CLA	1.000	0.992	1.193	0.0090	0.236
	HAc	1.000^b^	1.344^a^	1.374^a^	0.0011	< 0.001
	NaAc	1.000	1.161	1.111	0.0014	0.549
	NaBu	1.000	0.935	0.919	0.0088	0.839

^a,b^ Means with different letters are significantly different from each other (*P* < 0.05)

**Table 9:** Expression of lipid accumulation-related genes in cells treated with nutrients

Nutrients		Genes	Control	Low dose	High dose	SEM	*P*-value
Vitamins	VB9	PPARγ	1.000	1.250	1.684	0.1944	0.382
		FABP4	1.000	1.102	1.313	0.1241	0.621
	VC	PPARγ	1.000	1.853	1.871	0.1838	0.071
		FABP4	1.000	1.093	1.128	0.1018	0.890
	VD (1,25-VD)	PPARγ	1.000^a^	0.329^b^	0.317^b^	0.1484	0.047
		FABP4	1.000^a^	0.501^b^	0.379^b^	0.1052	0.011
	VE (α-tocopherol)	PPARγ	1.000	0.796	1.190	0.1397	0.656
		FABP4	1.000	0.812	0.928	0.0691	0.545
	VK1	PPARγ	1.000	1.027	1.456	0.1048	0.108
		FABP4	1.000	0.813	0.800	0.0866	0.579
	VK2	PPARγ	1.000	1.158	2.085	0.3210	0.232
		FABP4	1.000	1.256	1.409	0.2058	0.699
Amino acids (derivatives)	Arg	PPARγ	1.000	1.100	1.951	0.3204	0.152
		FABP4	1.000	0.869	1.125	0.1340	0.744
	GAA	PPARγ	1.000	0.912	1.341	0.0886	0.103
		FABP4	1.000^c^	1.254^b^	1.486^a^	0.0723	< 0.001
	Leu	PPARγ	1.000	1.104	1.462	0.1195	0.277
		FABP4	1.000	1.344	1.848	0.1616	0.085
	Lys	PPARγ	1.000	1.445	1.565	0.4422	0.824
		FABP4	1.000	0.875	0.890	0.0411	0.415
	Met	PPARγ	1.000	1.228	1.003	0.0821	0.455
		FABP4	1.000	1.274	1.114	0.0941	0.518
	NCG	PPARγ	1.000	2.130	3.977	0.2435	0.399
		FABP4	1.000	1.512	2.034	0.1555	0.695
	Tau	PPARγ	1.000	0.792	0.881	0.1200	0.906
		FABP4	1.000	0.913	0.989	0.0561	0.816
	Trp	PPARγ	1.000	0.754	0.856	0.1268	0.740
		FABP4	1.000	0.799	0.708	0.1166	0.623
Fatty acids (salts)	ALA	PPARγ	1.000	1.026	1.522	0.4562	0.597
		FABP4	1.000	1.054	0.838	0.0509	0.203
	LA	PPARγ	1.000^b^	2.538^a^	2.597^a^	0.3676	0.035
		FABP4	1.000	1.335	0.984	0.1365	0.497
	*c*9, *t*11-CLA	PPARγ	1.000	0.785	0.664	0.1301	0.565
		FABP4	1.000	0.786	0.877	0.1162	0.779
	HAc	PPARγ	1.000	1.050	1.376	0.0925	0.185
		FABP4	1.000	1.563	0.985	0.1447	0.183
	NaAc	PPARγ	1.000	0.961	1.121	0.0742	0.697
		FABP4	1.000	0.978	1.156	0.0684	0.557
	NaBu	PPARγ	1.000	0.794	0.504	0.2012	0.614
		FABP4	1.000	0.958	1.026	0.0531	0.888

^a,b^ Means with different letters are significantly different from each other (*P* < 0.05)

## Discussion

The skeletal muscle-derived SVF cells constitute a heterogeneous mixture of mesenchymal stem cells, muscle satellite cells, endothelial cells, pericytes, preadipocytes, fibroblasts, immune cells, etc. [65]. Using our 3D culture model, we identified nutrients regulating angiogenesis, a key process that is spatially and temporally associated with adipogenesis [66]. Our screening revealed that VE, Leu, LA, *c*9, *t*11-CLA, and NaAc promote intramuscular angiogenesis by upregulating VEGFA, a key angiogenic factor that activates endothelial cell proliferation, migration, survival, and new vessel formation [67]. VC upregulates, whereas VD and NaBu downregulate *VEGFR2*, a functional receptor of VEGFA, to regulate intramuscular angiogenesis. The pro-angiogenic effects of VC [68, 69], Lys [70], Tau [71], LA [72] and the inhibitory effects of VB9 [73], NaBu [74] on human umbilical vascular endothelial cells (HUVECs) align with our findings on sheep muscle SVF cells. In vivo studies further support our in vitro studies, as maternal supplementation with VE [75, 76], GAA [77], Met [78], and NCG [79] enhances placental angiogenesis and fetal development, indicating our 3D cell culture model accurately simulates early vascular development. Since blood vessels act as a progenitor pool for adipocyte replenishment [80], nutritional strategies that promote angiogenesis in skeletal muscle enhance the capacity of intramuscular fat deposition by increasing preadipocyte formation [32, 81]. The current study provided a list of pro-angiogenic nutrients that can be supplemented to livestock at the neonatal stage and the anti-angiogenic nutrients that need to be less supplemented or limited at the early stage when blood vessels and preadipocytes are formed to ensure maximal intramuscular fat deposition.

Adipogenesis from preadipocytes involves a transcriptional cascade initiated by ZFP423, which enhances BMP sensitivity in progenitors [82]. Subsequently, C/EBP-β induces the expression of C/EBP-α and PPARγ, these two master regulators mutually reinforce each other and activate terminal differentiation genes like *FABP4*, driving lipid accumulation [83]. In our screening, VC, Trp, and LA promote the formation of intramuscular preadipocytes by increasing the expression of *ZFP423*, whereas VE, VK1, VK2, GAA, Leu, ALA, and HAc primarily upregulate *PPARγ* expression and promote adipogenesis. In addition, GAA enhance fatty acid transport and metabolism in intramuscular adipocytes by upregulating *FABP4*. HAc may promote adipocyte differentiation and fatty acid synthesis by histone acetylation [84] or activation of G protein-coupled receptors (GPCR) [85]. Leu primarily functions as an activator of the mTOR signaling pathway, which is crucial for adipocyte differentiation and lipid metabolism [86]. Conversely, VB9, VD, Lys, and NaBu inhibited the expression of *PPARγ*. Studies have shown that 1,25-VD binds VDR to form a heterodimer with the retinoid X receptor (RXR), regulating transcription of the target genes through vitamin D response elements (VDRE) located in the promoter region [87]. Since PPARγ also heterodimerizes with RXR to regulate adipogenic genes [88], competition between VDR and PPARγ may underlie VD’s anti-adipogenic effects. Although Lys deprivation suppresses adipogenesis in 3T3L1 cells [89], our results agree with studies in livestock animals [32, 81] and primary cells [90, 91].

Most animal studies on IMF deposition have focused on the finishing phase. For instance, Leu supplementation during the fattening period increased IMF in pigs without affecting carcass fat content [92], demonstrating depot-specific regulation. Similarly, VC [93, 94], Arg [95, 96], and NCG [97] increased IMF deposition in fattening cattle or pigs while reducing other fat depots. Lys [98] or Met restriction [99] also increased porcine IMF. However, these nutrients did not obviously affect lipid accumulation in sheep intramuscular adipocytes in the current study. These nutrients, especially amino acids, may regulate adipogenesis indirectly by targeting muscle development, suggesting a limitation of the current model due to the absence of muscle cell coculture. At the lipid accumulation stage, our invitro screening identified VE, VK1, GAA, Leu, LA, and HAc as promoters, and VD as an inhibitor. Thus, dietary levels of these promoters could be increased and VD restricted during the fattening stage to increase intramuscular fat accumulation.

The application of screened nutrients in livestock production requires further in vivo validation. It is worth noting that the supplemented nutrients may be degraded and ineffective in the rumen of ruminants in animal trials or farming production, so protective technologies for active compounds need to be employed, such as fat encapsulation, microencapsulation, and polymer coating [100]. Alternatively, some nutrients, such as vitamin A, can be administered through intramuscular injections [9, 11], while other nutrients may be supplemented through milk replacers during early lactation.

## Conclusions

This study identifies nutrients that modulate IMF deposition by targeting angiogenesis and adipogenesis in a 3D culture model. Pro-angiogenic nutrients (e.g., VC, VE, VK1, GAA, Leu, Lys, Met, NCG, Trp, ALA, LA, *c*9, *t*11-CLA, HAc, NaAc) enhance preadipocyte formation by promoting vascular development, while anti-angiogenic nutrients (e.g., VB9, VD, VK2, Tau, NaBu) restrict it. In addition, VC, VE, VK1, VK2, GAA, Leu, NCG, Trp, ALA, LA, and HAc promoted adipocyte differentiation, with VE, VK1, GAA, Leu, LA, and HAc further enhancing lipid accumulation. While nutrient screening offers a roadmap for optimizing marbling in meat production, particularly neonatal supplementation strategies, discrepancies between in vitro adipocyte responses and livestock trials highlight context-dependent effects. Further in vivo validation is essential to refine dietary interventions for enhancing IMF deposition during critical developmental stages in livestock.

## Data Availability

All data generated or analyzed during this study are available from the corresponding author upon reasonable request.

## Abbreviations

ALA: Alpha-linolenic acid
Arg: Arginine
*c*9, * t*11-CLA: *Cis*-9, *trans*-11 conjugated linoleic acid
GAA: Guanidinoacetic acid
HAc: Acetic acid
LA: Linoleic acid
Leu: Leucine
Lys: Lysine
Met: Methionine
NCG: N-carbamoylglutamic acid
NaAc: Sodium acetate
NaBu: Sodium butyrate
Tau: Taurine
Trp: Tryptophan
VB9: Vitamin B_9_
VC: Vitamin C
VD: Vitamin D
VE: Vitamin E
VK1: Vitamin K_1_
VK2: Vitamin K_2_
